# Advanced nasopharyngeal carcinoma radiotherapy with volumetric modulated arcs and the potential role of flattening filter-free beams

**DOI:** 10.1186/1748-717X-8-120

**Published:** 2013-05-14

**Authors:** Mingzan Zhuang, Tuodan Zhang, Zhijian Chen, Zhixiong Lin, Derui Li, Xun Peng, Qingchun Qiu, Renhua Wu

**Affiliations:** 1 Department of Radiation Oncology, Tumor Hospital of Shantou University Medical College, Shantou 515000, China; 2 Department of Medical Physics and Computer Application, Shantou University Medical College, Shantou 515000, China; 3 Department of Radiation, The Second Affiliated Hospital of Shantou University Medical College, Shantou 515000, China

## Abstract

**Purpose:**

The purpose of this study is to investigate the dosimetric characteristics of volumetric modulated arc therapy (VMAT) with flattening filter-free (FFF) beams and assess the role of VMAT in the treatment of advanced nasopharyngeal carcinoma (NPC).

**Methods:**

Ten cases of CT data were randomly selected from advanced NPC patients. Three treatment plans were optimized for each patient, RapidArc with FFF beams (RA-FFF), conventional beams (RA) and static gantry intensity-modulated radiation therapy (IMRT). The doses to the planning target volumes (PTVs), organs at risk (OARs), skin and normal tissue were compared. All the plans were delivered on a Varian TrueBeam linear accelerator and verified using the Delta4 phantom. Technical delivery parameters including the mean gamma score, treatment delivery time and monitor units (MUs) were also analyzed.

**Results:**

All the techniques delivered adequate doses to the PTVs. RA-FFF gave the highest *D*_1*% *_(dose received by 1% of the volume), but the poorest conformity index (CI) and homogeneity index (HI) among the PTVs except for the planning target volume of involved regional lymph nodes (PTV66) CI, which showed no significant difference among three techniques. For the planning target volume of the primary nasopharyngeal tumor (PTV70), RA-FFF provided for higher mean dose than other techniques. For the planning target volume receiving 60 Gy (PTV60) and PTV66, RA delivered the lowest mean doses whereas IMRT delivered the highest mean doses. IMRT demonstrated the highest percentage of target coverage and *D*_99*% *_for PTV60. RA-FFF provided for the highest doses to the brain stem, skin and oral cavity. RA gave the highest *D*_1*% *_to the right optic nerve among three techniques while no significant differences were found between each other. IMRT delivered the highest mean doses to the parotid glands and larynx while RA delivered the lowest mean doses. Gamma analysis showed an excellent agreement for all the techniques at 3%/3mm. Significant differences in the MUs were observed among the three techniques (p < 0.001). Delivery times for RA-FFF and RA were 152 ± 7*s *and 153 ± 7*s*, respectively, nearly 70% lower than the 493 ± 24*s *mean time for IMRT.

**Conclusions:**

All treatment plans met the planning objectives. The dose measurements also showed good agreement with computed doses. RapidArc technique can treat patients with advanced NPC effectively, with good target coverage and sparing of critical structures. RA has a greater dosimetric superiority than RA-FFF.

## Introduction

Radiotherapy treatment for nasopharyngeal carcinoma (NPC) is often difficult due to the location of several tumor-adjacent organs at risk (OARs) including the brain stem, spinal cord, parotid glands and optic nerves. The application of sophisticated techniques is required to minimize the risk of toxicity or to adequately deliver curative doses. RapidArc is based on the volumetric modulated arc therapy (VMAT) technique and is developed to simultaneously optimize the multi-leaf collimator (MLC) shape, dose rate and gantry angle. It can obtain a dose distribution similar to the static gantry intensity-modulated radiation therapy (IMRT). Numerous studies have previously compared the dose distribution of VMAT and IMRT, and generally suggested that VMAT provides rapid, safe and accurate radiotherapy for many tumors, such as lung, gliomas, brain metastases, and some head and neck cancers [1-5].

TrueBeam (Varian Medical Systems, Palo Alto, CA) is a new linear accelerator designed to deliver flattened, as well as flattening filter-free (FFF) beams. The bremsstrahlung distribution from photons in the MeV energy range is strongly forward peaked and demonstrates both an energy and intensity variation of the primary photon fluence with emission angle. To compensate for this variation, the flattening filter has been introduced in the treatment head of a medical accelerator, which results in an almost uniform dose at a certain depth. With the development of IMRT techniques in all its forms, the flattening filter in many cases becomes redundant because the MLC can be used to reach the desired dose distribution.

There is an increasingly interest in the clinical usage of FFF beams. It was found that RapidArc with FFF beams results in minor improvements in plan quality with the potential for additional useful reduction in the treatment time for advanced esophageal cancer [6]. Subramaniam et al. [7] also reported that for chest wall radiotherappy, RapidArc with FFF beams showed the possibility to further reduce the dose delivered to healthy tissue compared to RapidArc with conventional flattened beams, suggesting their applicability for large and complex targets. With FFF beams the dose rate is increased up to 1400 monitor unit (MU)/min for the 6MV beam. The removal of flattening filter was also shown to reduce of out-of-field dose due to the reduction of head scatter and residual electron contamination. This leads to a possible faster treatment with reduced out-of-field dose exposure [8,9]. The main purpose of the present study is to assess the role of FFF beams in reducing the doses to the OARs while preserving adequate target coverage. It has been previously demonstrated that for medium and small size targets, FFF beams might be suitable for IMRT planning and that the out-of-field dose could be significantly reduced, resulting in better OAR protection [10]. It is important to demonstrate whether these advantages could be extended to large targets in a complex anatomic situation such as NPC. The second objective is to determine whether VMAT can be effective in the treatment of advanced NPC, confirming the data obtained by other groups [11-13].

## Materials and methods

### Patient characteristics

This study included 10 advanced NPC patients (median age 53 years, range 33–76 years) who had received radiotherapy continuously in the Radiation Oncology Department, Tumor Hospital of Shantou University Medical College. According to the stage of NPC (American Joint Committee on Cancer’s staging system 7th), the clinical stages of patients were as follows: stage III, 6 and stage IV, 4. Among these 10 patients, two patients had been diagnosed as T4 and two patients as N3.

### Delineation of target volumes and OARs

All target volumes were delineated slice by slice on the treatment planning CT images according to the International Commission on Radiation Units and Measurements Report 83 guidelines [14]. Gross tumor volume (GTV) was defined as the gross extent of the tumor shown by CT/MRI imaging, and this included the primary nasopharyngeal tumor (GTV70) as well as all involved regional lymph nodes (GTV66). Clinical target volume (CTV) was defined as the GTV plus a 5–10 mm margin for potential microscopic spread, including the regional lymph node draining areas. Planning target volumes (PTVs), which include PTV70, PTV66 and PTV60, were generated by 3mm outer margin of GTV70, GTV66 and CTV, respectively. The PTV66 and PTV60 were also generated by 3 mm apart from the surface of the body to avoid the parts extended to the outside of the body and the build-up effect. The median volumes for PTV70, PTV66 and PTV60 were 51±27*cm*^3^, 66 ± 33*cm*^3^, and 537 ± 137*cm*^3^, respectively. The lengths of the targets for all patients were > 20cm. The OARs, including the spinal cord, brain stem, lens, optic nerves, parotid glands, oral cavity and larynx, were contoured following anatomic definitions. Normal tissue was defined as the body volume subtracted by all the PTVs and OARs, and the skin was defined as the ring generated by the 7mm inter margin of the body.

### Treatment plan management

Three treatment plans were optimized for each patient on the CT imaging, RapidArc with FFF beams (RA-FFF), RapidArc with conventional flattened beams (RA) and static gantry IMRT. The IMRT plans were generated using sliding window dynamic delivery with conventional beams.

All RapidArc plans and IMRT plans were optimized on the Eclipse treatment planning system (Varian Medical Systems, Palo Alto, CA), using 6MV or 6MV FFF beams from a TrueBeam linear accelerator (Varian Medical Systems, Palo Alto, CA). Considering the large target volumes of advanced NPC and surrounding complex OARs, two arcs were adopted for both RapidArc plans and the coplanar fixed 9-field plan, seperated at 40° apart, was selected for IMRT. For RapidArc, the collimator rotation was set at 30° and two coplanar arcs were delivered with opposite rotation (clockwise and anticlockwise). The maximal dose rate was set to 600 monitor units (MU)/min for RA and 1400 MU/min for RA-FFF. For IMRT, a fixed dose rate of 600 MU/min was applied and the collimator rotation was set at 0°.

The accelerator was calibrated to deliver 0.01Gy/MU to water at a depth of maximal dose for a 10 × 10cm field at a source-to-surface distance of 100 cm following the American Association of Physicists in Medicine Task Group 51 report [15]. Optimization methods and parameters were similar for all patients across all three techniques. The progressive resolution optimizer algorithm (version 10.0.28) was used for RapidArc optimization and the dose volume optimizer algorithm (version 10.0.28) for IMRT. The planning objectives for the target volumes and OARs used in this study are listed in Table 1. For all patients, the dose volume constraints were defined to match the planning objectives and the normal tissue objective automatic tool was activated to minimize the dose spread outside the PTVs. Volumetric doses were calculated using the anisotropic analytical algorithm (version 10.0.28) with a dose calculation grid of 2mm. The simultaneous boost plan was used and the prescribed doses were 70 Gy to PTV70, 66 Gy to PTV66 and 60 Gy to PTV60 in 31 fractions. Each plan was normalized to meet the same objectives with 70 Gy covering 95% of the PTV70.

**Table 1:** The planning objectives in both RapidArc and IMRT plans for NPC

**Structures**	**Planning objectives**
PTV70	V70 ≥ 95*%*,*D*_1*% *_≤ 77Gy
PTV66	V66 ≥ 95%
PTV60	V60 ≥ 95 %
Brain stem	*D*_1*%*_ < 60Gy
Spinal cord	*D*_1*%*_ < 45Gy
Optic nerves	*D*_1*%*_ < 50Gy
Lens	*D*_1*%*_ < 10Gy
Larynx	Dmean < 40Gy
Oral cavity	Dmean < 40Gy
Parotid glands	Dmean < 40Gy
Normal tissue	as low as possible

Abbreviations: PTV70 = planning target volume of the primary nasopharyngeal tumor; PTV66 = planning target volume of involved regional lymph nodes; PTV60 = planning target volume receiving 60 Gy; ***V***_***D***_ = the percentage volume of the PTV at the prescribed dose and D was the prescribed dose; ***D***_***1%***_ = dose received by 1% of the volume.

Quantitative evaluation of the plans was performed using the mean of dose-volume histograms (DVHs). For the PTVs, *D*_99*%*_ and *D*_1*%*_ (dose received by 99% and 1% of the volume), mean dose, target coverage (*V*_*D*_), conformity index (CI), and homogeneity index (HI) were compared. *V*_*D*_ was the percentage volume of the PTV at the prescribed dose and D was the prescribed dose. CI was calculated using the equation: *CI* = (*PTV*_*ref*_ ÷ *V*_*PTV*_) × (*PTV*_*ref*_ ÷ *V*_*ref*_), where *PTV*_*ref*_ represented the volume receiving the prescription dose in the target volume, *V*_*PTV*_ stood for the volume of the PTV, and *V*_*ref*_ was the volume that received the prescribed dose. HI was evaluated as the difference between *D*_1*%*_ and *D*_99*%*_ divided by the prescription dose [16-18]. For OARs, *D*_1*%*_ was applied to evaluate the doses to the brain stem, spinal cord, optic nerves, and lens, and the mean dose was applied to evaluate the doses to parotid glands, larynx, oral cavity, normal tissue and skin. The treatment delivery time (including machine preparation) and the MUs of the three techniques were also compared. The RapidArc and IMRT fields were connected to each other as doable with TrueBeam.

### Dose verification

Phantom dose verifications were also performed for all the plans using the Delta4 (ScandiDos, Uppsala, Sweden). It is a cylindrical phantom for patient specific quality assurance. Absorbed dose was measured in two orthogonal detector arrays. The spacing between detectors is 5 mm in the center (8 × 8*cm*^2^) and 10 mm outside (20 × 20*cm*^2^). The measured dose planes were compared to the ones computed with a criterion of 3% and 3mm. A detector point was considered to pass if the calculated gamma index (GI) was smaller than 1. Points with less than 10% of the maximum dose were not taken into account.

### Statistical analysis

SPSS 11.0 software (IBM, Chicago, IL) was applied for statistical data management and analysis. To determine statistical significance, the non-parametric Friedman and post hoc Wilcoxon tests were performed with p-values < 0.05 considered to be significant. The data were presented as the averages over all patients and error was the one standard deviation level.

## Results

**Figure 1:**
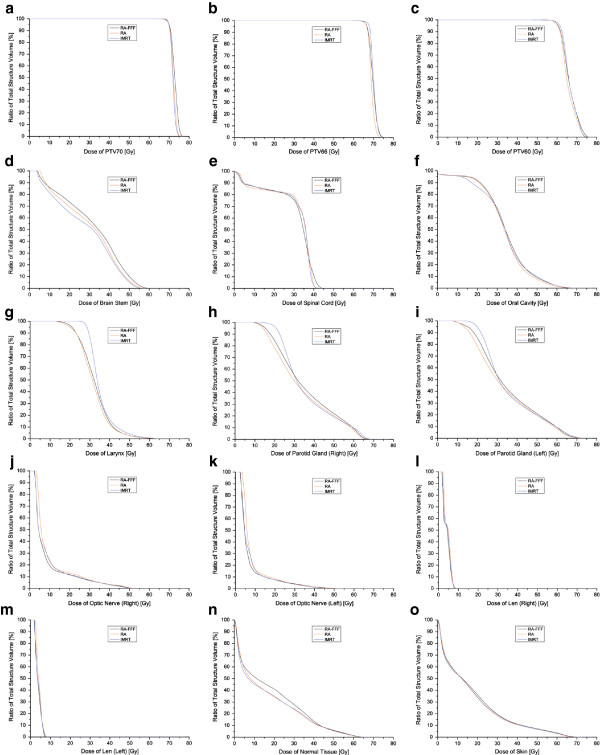
The dose distributions for three techniques.

**Figure 2:**
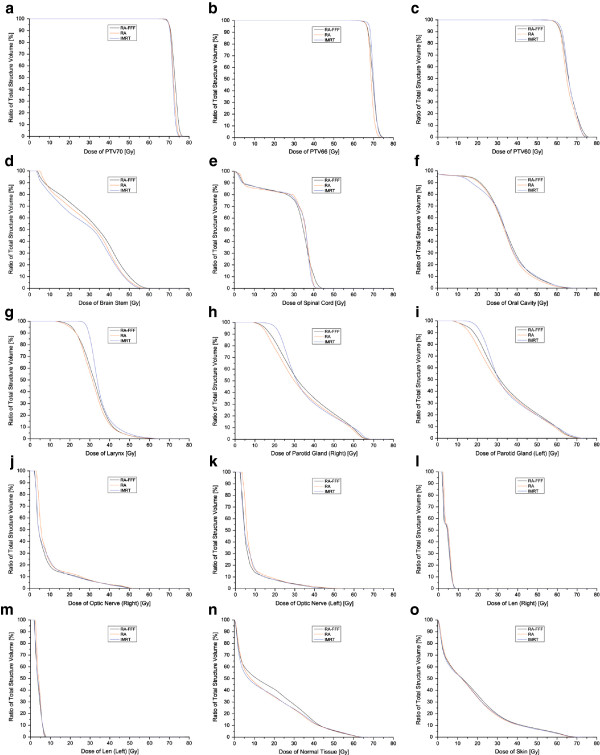
Dose-volume histograms for three techniques.

All 10 cases in the study received RA-FFF treatment planning, with a prescribed dose of 70*Gy* to 95% of the PTV70, resulting in a mean PTV70 dose of 72.80 ± 0.72*Gy*. Corresponding RA and IMRT treatment planning resulted in a mean PTV70 dose of 72.15±0.68*Gy*, 71.95 ± 0.21*Gy*, respectively. The dose distributions for the three plans are shown for 1 patient in Figure 1. Figure 2 presents the average DVHs for the PTVs and OARs for the entire group of patients.

Table 2 lists the statistical data of the PTVs for all studied cases. All techniques met the planning requirement for delivering the prescribed dose to at least 95% of the PTVs, and IMRT demonstrated the highest percentage of target coverage (p = 0.002) and *D*_99*%*_ (p = 0.006) for PTV60. RA-FFF provided for higher *D*_1*%*_ than other techniques and gave the poorest CI and HI for the PTVs except for the CI for PTV66, which showed no significant difference among the three techniques. For PTV70, RA-FFF also provided for higher mean dose than other techniques (p = 0.007). For PTV60 and PTV66, RA delivered the lowest mean doses, which were closer to the prescribed doses, whereas IMRT delivered the highest mean doses. No significant difference among the different techniques could be established in other PTV parameters.

**Table 2:** Comparison of PTV doses among the three techniques

**Target**	**RA-FFF**	**RA**	**IMRT**	**p value**	***p***^**1**^	***p***^**2**^	***p***^**3**^
PTV70							
*D*_99*%*_(Gy)	68.52 ± 0.46	68.69 ± 0.59	69.06 ± 0.22	0.122	—	—	—
*D*_1*%*_(Gy)	75.40 ± 1.32	74.08 ± 1.12	74.30 ± 0.62	0.007	0.005	0.028	0.878
Dmean(Gy)	72.80 ± 0.72	72.15 ± 0.68	71.95 ± 0.21	0.007	0.005	0.012	0.333
V70(%)	95.00 ± 0.00	95.00 ± 0.00	95.00 ± 0.00	—	—	—	—
CI	0.486 ± 0.213	0.609 ± 0.199	0.519 ± 0.220	0.014	0.007	0.445	0.037
HI	0.087 ± 0.023	0.067 ± 0.021	0.069 ± 0.011	0.008	0.005	0.028	0.646
PTV66							
*D*_99*%*_(Gy)	65.26 ± 0.99	65.19 ± 0.98	66.07 ± 1.09	0.121	—	—	—
*D*_1*%*_(Gy)	72.74 ± 1.58	71.34 ± 1.46	72.25 ± 1.53	0.008	0.008	0.314	0.799
Dmean(Gy)	69.67 ± 0.99	68.90 ± 0.85	70.18 ± 1.18	0.001	0.008	0.155	0.008
V66(%)	97.65 ± 1.28	97.53 ± 1.37	99.08 ± 0.60	0.050	—	—	—
CI	0.209 ± 0.074	0.244 ± 0.074	0.226 ± 0.083	0.169	—	—	—
HI	0.108 ± 0.054	0.088 ± 0.052	0.080 ± 0.048	0.001	0.008	0.008	0.123
PTV60							
*D*_99*%*_(Gy)	57.97 ± 0.84	57.88 ± 0.77	58.54 ± 0.71	0.006	0.508	0.009	0.009
*D*_1*%*_(Gy)	74.31 ± 1.08	73.38 ± 1.02	73.30 ± 0.61	0.025	0.009	0.022	0.799
Dmean(Gy)	66.23 ± 0.93	65.55 ± 1.08	66.47 ± 0.91	0.001	0.005	0.333	0.005
V60(%)	96.36 ± 0.87	96.13 ± 0.95	97.88 ± 0.44	0.002	0.445	0.005	0.007
CI	0.793 ± 0.025	0.811 ± 0.023	0.813 ± 0.025	0.002	0.005	0.022	0.575
HI	0.230 ± 0.040	0.217 ± 0.044	0.199 ± 0.038	0.001	0.047	0.005	0.005

Abbreviations: CI = conformity index; HI = homogeneity index; ***D***_***99%***_ = dose received by 99% of the volume; ***p***^***1***^ = Wilcoxon test p value between RA-FFF and RA plans; ***p***^***2***^ = Wilcoxon test p value between RA-FFF and IMRT plans; ***p***^***3***^ = Wilcoxon test p value between RA and IMRT plans. Other abbreviations as in Table 1.

There was no significant difference in *D*_1*%*_ delivered to the spinal cord, lens and left optic nerve (Table 3). Each technique also provided similar mean dose to normal tissue of patients included in this study. RA-FFF provided for higher *D*_1*%*_ to the brain stem (p = 0.014) and higher mean doses to the oral cavity (p = 0.025) and skin (p = 0.008) compared to other techniques. RA gave the highest *D*_1*%*_ to the right optic nerve among three techniques (p = 0.007) while no significant differences were found between each other in post hoc Wilcoxon tests. IMRT delivered the highest mean doses to the parotid glands and larynx while RA delivered the lowest mean doses.

**Table 3:** Comparison of OAR doses among the three techniques

**OARs**	**RA-FFF(Gy)**	**RA(Gy)**	**IMRT(Gy)**	**p value**	***p***^**1**^	***p***^**2**^	***p***^**3**^
Spinal cord *D*_1*%*_	41.22 ± 2.51	39.58 ± 2.21	40.00 ± 1.46	0.061	—	—	—
Brain stem *D*_1*%*_	55.58 ± 3.25	53.22 ± 3.35	52.82 ± 2.52	0.014	0.013	0.017	0.359
Optic nerve (right) *D*_1*%*_	16.91 ± 22.14	17.93 ± 21.52	16.45 ± 19.50	0.007	0.059	0.959	0.059
Optic nerve (left) *D*_1*%*_	15.26 ± 18.32	16.55 ± 17.28	14.15 ± 15.75	0.061	—	—	—
Len (right) *D*_1*%*_	5.36 ± 2.65	5.74 ± 2.36	5.35 ± 2.54	0.236	—	—	—
Len (left) *D*_1*%*_	5.11 ± 2.08	5.32 ± 1.83	4.90 ± 1.95	0.232	—	—	—
Larynx Dmean	32.55 ± 2.97	31.83 ± 2.69	35.40 ± 1.90	0.001	0.025	0.007	0.005
Oral cavity Dmean	34.12 ± 2.23	33.31 ± 1.90	33.52 ± 1.38	0.025	0.017	0.169	0.646
Parotid (right) Dmean	35.95 ± 4.32	34.00 ± 3.87	36.57 ± 4.15	0.001	0.005	0.445	0.005
Parotid (left) Dmean	34.67 ± 3.62	33.47 ± 3.16	35.36 ± 2.97	< 0.001	0.005	0.059	0.005
Normal tissue Dmean	16.24 ± 2.10	16.41 ±2.09	16.20 ± 2.04	0.150	—	—	—
Skin Dmean	17.24 ± 2.31	16.82 ± 2.27	16.75 ± 2.32	0.008	0.017	0.007	0.646

Abbreviations: Abbreviations as in Table 2.

Table 4 lists the mean gamma score, delivery time and MUs, grouped by plantype. Patient-specific dose verifications were performed for 10 plans in three different techniques. All three techniques showed equally good gamma scores. Dose computation, as well as dose delivery, was equally accurate for RA-FFF, RA and IMRT delivery with gamma analysis performed at 3%/3mm. All the plans had a GI above 95%. The delivery times for RA-FFF and RA were 152 ± 7*s* and 153 ± 7*s*, respectively, which was reduced by nearly 70% compared to that of IMRT with the mean time of 493 ± 24*s*. The MUs were significantly different among the three techniques (p < 0.001).

**Table 4:** Comparison of GI, delivery time and MUs among the three techniques

**Parameters**	**RA-FFF**	**RA**	**IMRT**	**p value**	**p1**	**p2**	**p3**
GI	98.53*%* ± 1.02*%*	98.49*%* ± 1.28*%*	98.66*%* ± 1.04*%*	0.746	—	—	—
Time(s)	152 ± 7	153 ± 7	493 ± 24	< 0.001	0.539	0.005	0.005
MUs	536 ± 46	501 ± 25	1199 ± 129	< 0.001	0.022	0.005	0.005

Abbreviations: GI = gamma index; MUs = monitor units; Other abbreviations as in Table 2.

## Discussion

Radiotherapy of NPC is a challenging task because of the complex anatomy, with bones, surrounding OARs and air cavities all in need of consideraion. Novel radiotherapy techniques are able to effectively reduce the treatment side effects, while maintaining good local control. According to a study by White et al. [19] on the radiation doses in VMAT and IMRT for NPC, VMAT showed superior, or comparable, dose conformity and target coverage in the PTVs. VMAT plans also achieved significant improvements in dose reduction to OARs and a significant reduction in treatment delivery time for VMAT treatment technique was noted. It has also been indicated that given the similar target dose coverage, VMAT is able to treat NPC more efficiently with less damage to OARs [13]. Lee et al. [11] found that dual arc VMAT produced plans with similar target coverage, as well as sparing OARs, as compared to 7-field IMRT. VMAT outperformed IMRT by effectively reducing the delivery time.

However, these previous studies did not compare the difference between FFF beams and conventional beams in VMAT for NPC. FFF beams have several potential advantages, such as increased dose rate, reduced collimator scatter, reduced head leakage, and reduced out-of-field doses to the patient. This study was to assess the role of FFF beams in VMAT with a focus on advanced NPC. Based on dosimetric evaluation in this study, it can be found that all the treatment plans could meet the planning objectives, and the RapidArc technique can treat patients with advanced NPC effectively, with good target coverage and sparing of critical structures, results similar to those found by others [13,19]. RA-FFF showed poorer conformity and heterogeneity compared to other techniques. This suggests that FFF beams might be applicable for treatment of large targets in a complex anatomic situation, with adequate doses to the targets and RA was more likely to give lower dose to most OARs and achieve better conformity for the PTVs. We have also focused on dosimetric verification in this study. The evaluation of computed dose on Eclipse and delivered dose at TrueBeam measured by the Delta4 indicated an excellent agreement for all the techniques. Gamma analysis demonstrated that all the plans were verified, with >95*%* of the measured points meeting the 3%/3mm criterion.

Our results are in contrast to those observed in other studies involving FFF beams for large and complex targets. Nicolini et al. [6] and Subramaniam et al. [7] reported that RapidArc with FFF beams provides minor improvements in plan quality, suggesting their applicability for large and complex targets. However, these studies just included one target. The number of OARs (≤ 5) was less than that of this study. In this study, the target volumes of three different dose levels, PTV70, PTV66 and PTV60 were delivered in a simultaneous boost plan. The lengths of the targets are > 20cm and the targets are surrounded by many critical neural tissues and sensitive structures such as optic nerves, brain stem, oral cavity, and parotid glands. The RA superiority over RA-FFF is possibly due to the use of rather large field sizes and complex target volumes.

The VMAT advantage in shortening the treatment time compared to IMRT plans is well known. In this study, the average delivery time for RA-FFF and RA was 152 ± 7s and 153 ± 7s, respectively, which was nearly 70% shorter than that of IMRT. Furthermore, the dose rate for the FFF beams can be substantially higher than the conventional beams (1400 MU/min vs 600MU/min). However, no significant difference in the delivery time was found between RA-FFF and RA. Although high dose rate (more than 1000 MU/min) was observed in RA-FFF, it did not lead to improvements in the delivery time. This is because in our cases the delivery time is largely limited by the gantry rotation speed and leaf speed, not the dose rate. In the hypofractionated treatment for liver tumours, Mancosu et al. [20] found that RapidArc with FFF beams resulted to be feasible with short beam on time.

In this study RA-FFF tended to show a lower mean dose to normal tissue compared to RA. It appears possible to predict a reduction of out-of-field dose when FFF beams are used. This is mainly related to reduced head scattering and residual electron contamination. However, the MUs in the RA-FFF plans were always greater than in the RA plans. The reason is that FFF beam intensity decreases with the off-axis distance, which can be clearly observed in larger field (≥10×10*cm*^2^) open beam dose profiles. As a result, off-axis distance-dependent modulation is needed for delivering uniform doses to large target volumes, possibly leading to greater MUs. This will lead, at least partially, to cancellation of the potential advantages for FFF beams such as reduced head scatter and out-of-field doses.

The spectrum of a 6 MV FFF beam is typically softer because the flattening filter acts as a beam hardener. The different spectrum of unflattened beams is reflected in the depth dose distribution. Vassiliev et al. [21] found that the depth dose distribution of unflattened 6 MV beams was similar to that of conventional 4–5 MV beams. Due to the softer spectrum of FFF beams, a slightly higher dose to the skin can be expected. A mitigating factor to this is that the scattered radiation and electron contamination from the flattening filter are eliminated. Among the three techniques RA-FFF provided for the highest mean dose to the skin. This could be avoided by using higher energies, e.g., 8 MV instead of 6 MV [9].

The collimator angles were fixed at 0° for IMRT planning in our study. A published study by Deng et al. [22] demonstrated that the tongue-and-groove effect was clinically insignificant for multiple-field IMRT because of the smearing effect of individual fields and the collimator angle was set to 180° which played a fundamental role in smearing effect of tongue-and-groove. However, some degree of collimator rotation is usually carried out in VMAT to minimize the cumulative effects of the tongue-and-groove effect and interleaf transmission [23]. In our study a 30° collimator angle in RapidArc was chosen. Mans et al. [24] indicated that a better plan quality could be achieved using a collimator rotation between 20° and 30°. Clivio et al. [17], Vanetti et al. [25] and Cozzi et al. [26] also used a 30–45° collimator rotation in the VMAT planning.

## Conclusions

This study demonstrates that, in the treatment planning of advanced NPC, RapidArc technique can treat patients with advanced NPC effectively, with good target coverage and sparing of critical structures, and RA gave superior, or similar sparing of OARs than other techniques. All the treatment plans could meet the planning objectives. The dose measurements also showed good agreement with computed doses. Although RA-FFF shows the potential for treatment of advanced NPC patients with adequate target coverage and sparing of OARs, RA has a greater dosimetric superiority than RA-FFF, and further studies are still required to evaluate their clinical outcomes.

## Competing interests

The authors declare that they have no competing interests.

## Authors’ contributions

MZ, TZ and XP designed the study, performed the treatment plans and quality assurance, interpreted the results of the study, oversaw the project completion. ZC, ZL, DL, QQ and RW participated in preparing of the study and criticized the manuscript. MZ performed the statistical analysis and drafted the manuscript with TZ. All authors contributed to the scientific setup of the study and revised the manuscript critically and they have approved the final version of the manuscript.
